# Sensory, structural breakdown, microstructure, salt release properties, and shelf life of salt-coated air-dried yellow alkaline noodles

**DOI:** 10.1038/s41538-023-00183-5

**Published:** 2023-03-17

**Authors:** Shin-Yong Yeoh, Hui-Ling Tan, Lubowa Muhammad, Thuan-Chew Tan, Maizura Murad, Azhar Mat Easa

**Affiliations:** 1 Food Technology Division, School of Industrial Technology, Universiti Sains Malaysia, 11800, USM Penang, Malaysia; 2 School of Hospitality and Service Management, Sunway Universiti, 47500 Petaling Jaya, Selangor Malaysia; 3 Department of Food Innovation and Nutrition, Mountains of the Moon University, Fort Portal, Uganda; 4 Renewable Biomass Transformation Cluster, School of Industrial Technology, Universiti Sains Malaysia, 11800 USM Penang, Malaysia

**Keywords:** Biochemistry, Proteins, Carbohydrates

## Abstract

Salt reduction in food has been employed to improve public health. The effects of salt coatings on sodium content, sensory properties, structural breakdown, microstructure, salt release properties, and shelf life of yellow alkaline noodles (YAN) were evaluated. 15 g/dL resistant starch HYLON™ VII (HC) or 5% (v/v) Semperfresh™ (SC) with 10, 20, and 30 g/dL sodium chloride (NaCl) were used. HC-Na30 and SC-Na30 had the highest sodium content and came closest to commercial YAN in taste and saltiness perception. Structural improvement was demonstrated with HC-Na10 and SC-Na10 as both noodles required maximum work to be broken down. Moreover, SEM micrographs of these noodles showed a more compact and dense appearance with increased continuity of the matrix and fewer voids and hollows. However, ruptured surfaces were observed in noodles coated with 20 and 30% salt. The enhanced salt release from the coatings was demonstrated in an in vivo analysis, with the released salt occurring rapidly from HC and SC coatings. HC-Na10 and SC-Na10 noodles had a shelf life of more than 8 days when stored at 4 °C, which is longer than HC-Na0 and SC-Na0 noodles. Storage at 4 °C decelerated the microbiological growth, changes in pH and CIE L* values in salt-coated noodles than storage at 25 °. Thus, HC-Na10 and SC-Na10 could be suitable formulations to replace commercial YAN.

## Introduction

Noodles are a famous traditional staple food in several Asian countries such as China, Japan, Indonesia, Malaysia, and Thailand^1^. About 40% of wheat in Asia is utilised to produce various noodles^2^. Noodles are the second most popular food among Chinese people globally after bread^3^. Yellow alkaline noodles (YAN) are a favourite type of noodle made of wheat flour, water, common salt, and *kansui* with a unique taste and flavour, as well as yellow colour and firm elastic texture^2^. Hylon™ VII is a commercial mixture of resistant and unmodified high amylose corn starch containing approximately 70% amylose. It is a type of dietary fibre and is cheap. Consumers will accept it because the fine powder is white, and the sensory attributes (colour and taste) are better than traditional dietary fibre^4^. Its use has shown promising results as a film coating for breakfast cereals^5^, probiotics *Lactobacillus casei*, and *Bifidobacterium adolescentis*^6^. Semperfresh^TM^ is a mixture of sucrose polyesters, sodium carboxymethyl cellulose, and mono- and di-glycerides-based coating widely used in the fruit and vegetable industry. It is GRAS, odourless and tasteless. It postponed ripening and prolonged the shelf life of fruits such as plum fruits^7^ and citrus fruits^8^ by inhibiting moisture loss, allowing gas exchange between fruits and the environment and reducing ethylene synthesis^9^. Reducing bruising and shrinkage, weight loss, colour loss, and pressure on fruits and vegetables during storage is crucial.

Adding NaCl and *kansui* to flour enhances the dough’s gluten strength and rheological attributes, which improve the noodles’ textural properties, flavour, and shelf life^10^. Fu^11^ recommended 1–3% of flour weight NaCl in the noodle formulation, while Wu et al.^12^ proposed 0.5–1.7% of flour weight of *kansui*. The World Health Organization^13^ reported that most populations consume 9–12 g NaCl daily, surpassing the recommended quantity of NaCl intake, which is 5 g/ NaCl or 2 g sodium per day. Processed foods such as instant noodles contain significant amounts of salt^14^. According to the report on health effects and dietary risks in 195 countries from 1990 to 2017 published in 2019 by the Global Burden of Diseases, Injuries, and Risk Factors Study diet collaborators^15^, high salt consumption is the top diet-related risk factor for death from noncommunicable disease (NCD) and disability-adjusted life-years (DALYs) worldwide. Excessive dietary salt consumption contributes to approximately 3 million deaths and 70 million DALYs worldwide^16^. Increased dietary sodium intake is strongly linked to cardiovascular disease, hypertension, and kidney disease^17^. Therefore, a gradual and consistent salt decrease over time has been advocated by Antúnez et al.^18^ as a cost-effective approach to enhance public health. Any attempt to minimise salt levels in noodles should preserve noodle texture and consumer acceptability. However, Sun et al.^19^ reported that reducing salt in foods could significantly impact the processing properties, sensory quality, and shelf life. Thus, decreasing salt in food products is challenging without compromising their sensory or processing characteristics.

Several attempts were made to reduce salt in food, including replacing sodium with salt substitutes^14,20^, redesigning food structure to optimise sodium release and transport^19^, adding salt boosters to enhance saltiness perception, altering the physical properties of salt^21^, and regulating sodium absorption^22^. Improving NaCl release from the food matrix by applying a salt coating could reduce the loss of NaCl content. Instead of the conventional method^23^, NaCl is introduced to the surface of noodles by adding it during the mixing phase of the formulation. The salt coating on unsalted noodles allows consumers to perceive a similar saltiness as traditional noodles because it increases the sodium dissolution rate from the food matrix into saliva^24^. Furthermore, consumers tend to slurp the noodles with NaCl without chewing them thoroughly. Therefore, as observed with potato crisps, some NaCl is not perceived during consumption^25^. In the long term, NaCl is secretly reduced and consumers can enjoy low-salt noodles like traditional YAN without compromising the sensory saltiness and taste.

In previous work, salt-coated YAN was prepared by immersing it in a solution of high amylose (HC) and Semperfresh^TM^ (SC) containing a variety of NaCl between 10 and 30% (w/v) and the influence of salt coatings on the textural, handling, cooking, and sensory attributes of YAN was investigated^26^. The highest textural and mechanical parameters, sensory hardness, and springiness were observed in HC-Na10 and SC-Na10 noodles. This study hypothesised that salt coatings were broken down in the mouth at a higher rate during mastication, and salt release in the mouth was enhanced. It could yield salt-coated YAN (lower salt content) without affecting the taste and saltiness perception. The objective of this study was to evaluate the effect of salt coatings (HC or SC) on sensory properties, structural breakdown parameters, microstructure, salt release properties, and shelf life of air-dried YAN.

## Results and discussion

### Sodium content and sensory evaluation of cooked noodles

The sodium contents of cooked noodles are shown in Table 1. The order was HC-Na30 > SC-Na30 > HC-Na20 > SC-Na20 > HC-Na10 > SC-Na10 > HC-Na0 > SC-Na0. The highest sodium levels were in cooked HC-Na30 and SC-Na30 noodles (3253 and 3006 mg/kg, respectively). This order was expected since both HC-Na30 and SC-Na30 noodles contained the highest salt. The lowest salt in cooked HC-Na0 (450 mg/kg) and SC-Na0 (403 mg/kg) was introduced from the sodium ions derived from the *kansui* reagent consisting of sodium/potassium carbonate or bicarbonate or a mixture of both. All these salt compositions are subject to the manufacturers. The present study’s cooked noodles contained less sodium, i.e. less than 50%, than cooked commercial YAN (7307 mg/kg or ~1.75% w/w). The typical salt addition level in commercial YAN’s formulation is 1–3% of the flour weight. Depending on local preference, the highest added salt can be increased to 8% in udon and hand-made noodles^11^.

**Table 1:** Final salt content of cooked noodles and sensory parameters for different types of noodles.

Samples	Final Na in cooked noodles (mg/kg)	Taste	Saltiness
HC-Na0	450 ± 33.94^e^	3.77 ± 0.77^c^	2.37 ± 0.8^e^
HC-Na10	1064 ± 54.07^d^	4.47 ± 0.85^b^	3.9 ± 0.54^c^
HC-Na20	2108 ± 142.24^c^	4.7 ± 0.86^ab^	4.7 ± 0.69^b^
HC-Na30	3253 ± 136.85^a^	5.2 ± 0.75^a^	5.6 ± 0.71^a^
SC-Na0	403 ± 45.45^d^	3.73 ± 0.44^c^	2.1 ± 0.65^e^
SC-Na10	962 ± 15.44^d^	4.4 ± 0.61^b^	3.13 ± 0.72^d^
SC-Na20	1942 ± 105.54^c^	4.7 ± 0.69^ab^	4.5 ± 0.76^b^
SC-Na30	3006 ± 140.95^b^	5.13 ± 0.85^a^	5.47 ± 0.81^a^
YAN	7307 ± 527.79	5.27 ± 0.81	5.77 ± 0.76

Results display mean values ± standard deviation (*n* = 3) values. Different lowercase letters indicate significant differences (*P* < 0.05) between values within a particular column. YAN was used as a reference and not included in the statistic.

A sensory evaluation was conducted to relate salt content and sensory perception (Table 1). The rate of salt release during the first few seconds of chewing defines perceived saltines^27^. Salt concentration made a significant difference in taste and saltiness (*P* < 0.05). The salt reduction should not adversely influence a product’s characteristic flavour and mouthfeel. The order of taste and saltiness perceptions were similar for both HC and SC noodles, showing increasing preferences with increasing salt coatings. HC-Na30 and SC-Na30 noodles showed the highest values, while HC-Na0 and SC-Na0 showed the lowest values (*P* < 0.05). In this study, salt concentration affected the saltiness perception in the noodles. Gulia et al.^28^ reported that NaCl improved the texture and flavour of noodles. The taste and saltiness scores of HC-Na0 and SC-Na0 were significantly lowest (*P* < 0.05). Salt has a significant impact on the palatability of foods. The increase in saltiness can enhance the overall taste, taste, and perception of aromatic components and mask bitterness^29^. Coatings without salt decreased the taste and saltiness of the noodles, and panellists did not favour these noodles. The salt coatings did not adversely impact the sensory attributes of the noodles.

### Structural breakdown analysis

A direct connection between structural breakdown properties and salt coatings reflects the significance of these properties to coated noodles. A complete extrusion cycle consists of downward and upward extrusion movements, and the structural breakdown patterns of the noodles can be demonstrated through 20 extrusion cycles. The area under each downward extruding motion (*Work 1*st) represents the work required to break down the whole noodle. The work done during the first extrusion cycle (*w*_*1*_) shows the total work needed to break down the intact noodles^30^.

The significant parameters derived from the decay curves are listed in Table 2. The total *Work 1*st was in order: HC-Na10, SC-Na10 > HC-Na20, SC-Na20 > HC-Na30, SC-Na30 > HC-Na0, SC-Na0. The salt concentration had a significant influence on the structural breakdown analysis. HC-Na10 and SC-Na10 noodles necessitated the most quantity of work (P < 0.05) to break down the integral structure (*Work 1*st), mostly due to the existence of salt on the outer surface of the noodles, which makes the surface harder and firmer. HC-Na10 and SC-Na10 noodles were the most elastic, hardest, firmest, and most easily degradable. These results agreed well with the mechanical and TPA analysis outcomes from previous studies, in which both noodles exhibited the highest mechanical and TPA parameters^26^.

**Table 2:** Structural breakdown parameters using MEC analysis for different types of noodles.

Samples	*Work 1*st (J)	w_inf_ (J)	*w*_*1*_ (J)	*n*_*1*_ (J/cycle)
HC-Na0	1.91 ± 0.04^c^	0.44 ± 0.02^a^	1.2 ± 0.05^c^	3.9 ± 0.02^d^
HC-Na10	2.46 ± 0.13^a^	0.4 ± 0.04^abc^	1.61 ± 0.07^a^	4.9 ± 0.05^a^
HC-Na20	2.34 ± 0.05^b^	0.37 ± 0.03^bcd^	1.57 ± 0.02^a^	4.77 ± 0.03^b^
HC-Na30	2.18 ± 0.08^c^	0.34 ± 0.03^cd^	1.46 ± 0.04^b^	4.62 ± 0.03^c^
SC-Na0	1.86 ± 0.08^c^	0.41 ± 0.03^ab^	1.19 ± 0.04^c^	3.89 ± 0.04^d^
SC-Na10	2.41 ± 0.06^a^	0.37 ± 0.02^abcd^	1.6 ± 0.03^a^	4.87 ± 0.02^a^
SC-Na20	2.3 ± 0.06^b^	0.34 ± 0.02^bcd^	1.55 ± 0.03^a^	4.69 ± 0.04^b^
SC-Na30	2.12 ± 0.08^c^	0.31 ± 0.02^d^	1.44 ± 0.05^b^	4.54 ± 0.03^c^
YAN	0.98 ± 0.02	0.25 ± 0.02	1.17 ± 0.04	3.04 ± 0.04

Results display mean values ± standard deviation (*n* = 3) values. Different lowercase letters indicate significant differences (*P* < 0.05) in values between samples for each MEC parameter. YAN was used as a reference and not included in the statistic.

The work done during the first extrusion cycle (*w*_*1*_) shows how much work was crucial to break down the intact noodle in the following order HC-Na10, SC-Na10 > HC-Na20, SC-Na20 > HC-Na30, SC-Na30 > HC-Na0, SC-Na0. A higher value of *w*_*1*_ represents additional work required to deform and break the structures. All samples showed significant differences (*P* < 0.05). Significantly, HC-Na10 and SC-Na10 noodles had the highest *w*_1_ values (P < 0.05). Commercial YAN has a lower w_1_ value than all samples. After several extrusions (*w*_inf_), no additional structure element degraded. At this point, the noodle strands turned semi-solid without further structural breakdown. The *w*_*1*_ results agreed well with the TPA and tensile strength outcomes from a previous study^26^, whereby more work was crucial to break down the hardest structure and highest tensile strength. The *w*_*1*_ value of commercial YAN was lower than that of noodles, suggesting that less work is essential to break down its structure. It could be owing to their shape, size, and composition. HC-Na0 noodle yielded the highest gain (*P* < 0.05), indicating that the HC-Na0 showed sticky behaviour after extrusion. However, the *w*_inf_ value of HC-Na0 was higher than that of commercial YAN. No salt was employed in this formula to promote protein–protein interactions and the formation of a strong gluten network, consequently causing stickiness. Avramenko et al.^31^ reported that salt was utilised to prevent dough stickiness. Moreover, the addition of artificial saliva facilitated the breakdown of starch and increased stickiness. The enzymes in the artificial saliva digested the starch granules in the starch coating of HC-Na0 noodles. The parameter *n*_*1*_ reflects the degradation rate with an increasing multitude of extrusions. Lower *n*_*1*_ indicates a higher breakdown rate, implying that samples break down quickly. HC-Na0 and SC-Na0 noodles significantly scored the lowest *n*_*1*_ values (*P* < 0.05), suggesting that the breakdown rates of noodles with zero salted coatings were higher than those of noodles with salted coatings. HC-Na10 and SC-Na10 significantly illustrated the highest *n*_*1*_ values (*P* < 0.05). It could be owing to their harder and firmer structure. All noodles demonstrated higher *n*_*1*_ values compared to commercial YAN. According to Fan et al.^2^, *kansui* improved noodles’ hardness, chewiness, and tensile strength. A positive correlation exists between increased covalent protein cross-linking of noodles made with *kansui* and increased noodle hardness^32^.

Na^+^ is a structure-making or salting-out ion with a smaller or more symmetrical structure^33^. It promotes hydrogen bonds between starch molecules and prevents interaction between starch and water molecules, resulting in reduced starch swelling and solubility. Therefore, it increases the gelatinisation temperature and enthalpy^34^. Li et al.^35^ reported that NaCl in a specific concentration range (1–4 mol/L) inhibited the gelatinisation of corn starch. Furthermore, lower concentrations (1 and 2 M) of NaCl raised the peak gelatinisation temperature of corn starch, while higher concentrations (3 and 4 M) decreased its peak gelatinisation temperature. Li et al.^36^ found that increasing the lower NaCl concentration increased the gelatinisation degree. However, all NaCl concentrations (1–4 M) inhibited the A-type polymorph in Chinese yam starch. The inhibitory effect of low NaCl concentration contributed to the dominant Na^+^ water structure-making effect, while the electrostatic interaction between starch –OH groups and Na^+^ ions was significant at high concentrations. Both coatings could carry a quantity of salt that impacted the overall quality characteristics of the cooked noodles.

### Digital microscope image analysis

**Fig. 1:**
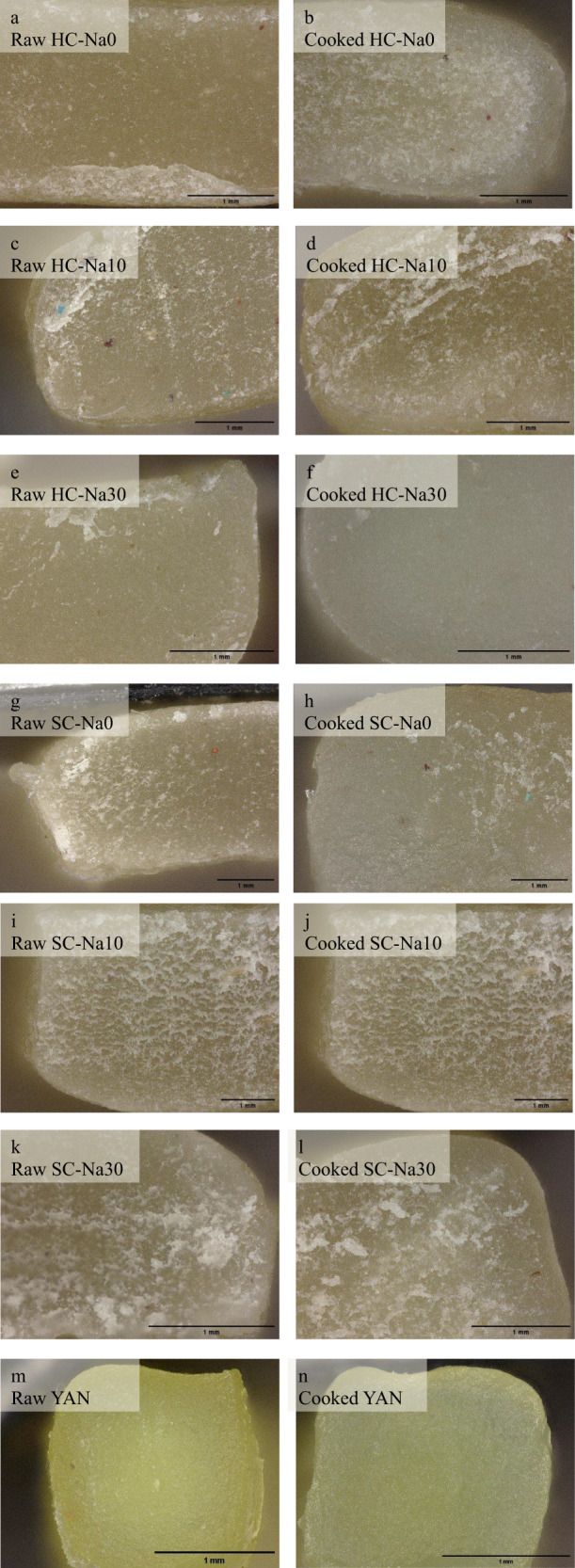
Digital microscope images of noodle samples at 100× magnification consisting of raw and cooked HC, SC, and YAN noodles. Scale bar = 1 mm. **a** Raw HC-Na0, **b** cooked HC-Na0, **c** raw HC-Na10, **d** cooked HC-Na10, **e** raw HC-Na30, **f** cooked HC-Na30, **g** raw SC-Na0, **h** cooked SC-Na0, **i** raw SC-Na10, **j** cooked SC-Na10, **k** raw SC-Na30, **l** cooked SC-Na30, **m** raw YAN, **n** cooked YAN.

Figure 1 shows the digital microscope images of raw and cooked fresh noodle samples at 100× magnification consisting of HC-Na0, HC-Na10, HC-Na30, SC-Na0, SC-Na10, SC-Na30, and commercial YAN. Coatings were observed outside the yellowish layer of all raw HC and SC noodles. The coatings were observed on the surface of the noodles after cooking. YAN was more yellowish than all HC and SC noodles. It was probably due to the different amounts of *kansui* reagent used in its formulation. The yellow colouring of YAN results from the naturally occurring flavones in flour, namely apigenin-C-diglycosides. These compounds appear colourless under acidic or neutral pH and become yellow under alkaline pH^37^. Initially, they are only found in wheat germ and are relatively stable at ambient temperature^38^.

### Microstructure analysis

**Fig. 2:**
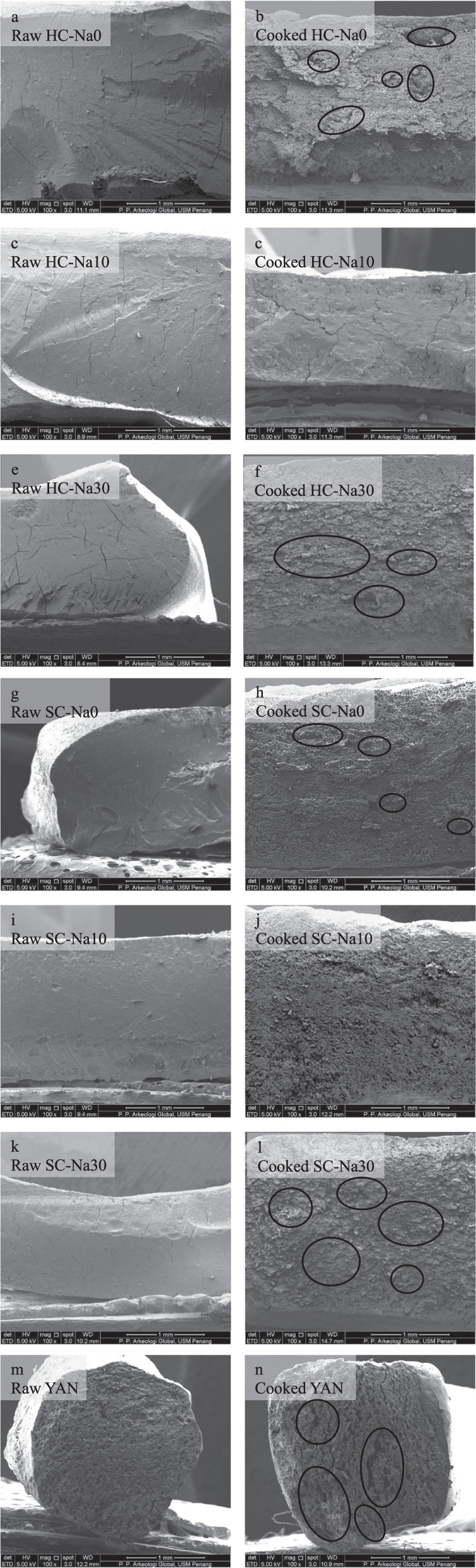
SEM images of noodle samples at 100× magnification consisting of raw and cooked HC, SC, and YAN noodles. The circles in the images represent larger hollows and voids. Scale bar = 1 mm. **a** Raw HC-Na0, **b** cooked HC-Na0, **c** raw HC-Na10, **d** cooked HC-Na10, **e** raw HC-Na30, **f** cooked HC-Na30, **g** raw SC-Na0, **h** cooked SC-Na0, **i** raw SC-Na10, **j** cooked SC-Na10, **k** raw SC-Na30, **l** cooked SC-Na30, **m** raw YAN, **n** cooked YAN.

The optimum cooking time was applied to all noodles. Cooking caused alterations in the structure of noodles. After cooking, noodles were seen with hollow structures triggered by heat and water absorption. Larger hollows and voids were more apparent in the noodles without salt (Fig. 2b, h), likely resulting from swollen and ruptured starch granules in the gluten network. These starch granules increased water penetration into the central core of the noodles, leading to the highest cooking yield and cooking loss^26^. In addition, the lowest mechanical and textural parameters were potentially related to their microstructure. Ruptured surfaces were discovered in noodles coated with 30% salt (Fig. 2f, l). High levels of NaCl (30%) destroyed the structure of noodles. Similar findings were reported by Tan et al.^39^, where high NaCl concentration damaged the structure of the noodles, as the porous nature of high salt noodles facilitated water penetration into the noodle core and allowed excellent heat/mass transfer. Noodles coated with 10% salt (Fig. 2d, j) have a denser appearance with an expansion in the matrix continuity and fewer hollows, contributing to the lowest cooking yield reported by Yeoh et al.^26^. Furthermore, they stated that the continuity of the gluten network of these noodles yielded the highest mechanical and textural properties. NaCl promoted the formation of thread-like or fibrous gluten structures and a firmer and more resistant network, similar to Li et al.^32^. They also pointed out that salted noodles had a more coherent and smoother surface than noodles without salt.

Interestingly, a study by Fan et al.^2^ showed that *kansui* formed a membrane-like substance in the microstructure of noodles, possibly due to the rapid formation of firm gluten networks. They also demonstrated that the coherence of the sample’s protein network structure showed improvement with the presence of *kansui*. This was possibly due to the increased levels of disulfide bonds, ordered structures in protein molecules, and cross-linking between molecules in noodles by *kansu*i.

Overall, a 10% salt coating induced a structural enhancement in the noodles, resulting in a firmer texture that could reduce structural destruction caused by cooking. For this reason, maximum work was critical to break noodles with 10% salt coating during structural breakdown analysis (Table 2). HC-Na10 and SC-Na10 also exhibited the highest breakdown rate and *n*_1_ values. More extensive hollows and voids with a reduction in the continuity of the noodle’s matrix were discovered in the cooked YAN (Fig. 2n).

### Salivary conductivity and pH

#### Salivary conductivity

According to Kuo and Lee^40^, food is broken down into smaller pieces and mixed with saliva during mastication. The food particles are moistened, softened, and combined with digestive enzymes to form a swallowable bolus. Mastication can reduce the size of food particles by chewing for a standardised time and the required number of chews to ensure the food is ready to eat. Chewing leads to increased salivary secretion owing to the activation of the masticatory-salivary reflex. They produce a watery, protein-rich fluid with high levels of enzymes like α-amylase.

**Fig. 3:**
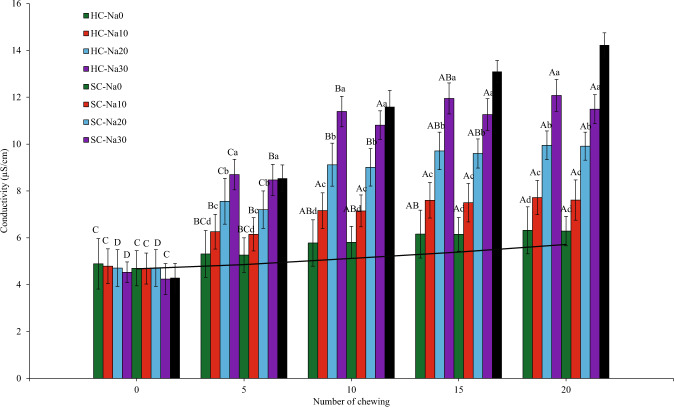
Saliva conductivity after chewing different types of noodles at 0, 5, 10, 15, and 20 chews. Error bars indicate mean values ± standard deviations (*n* = 10) values. [—] Saliva conductivity after chewing no noodles (blank) with a fake chewing action. Different lowercase letters indicate significant differences (*P* < 0.05) between samples for each number of chewing. Different uppercase letters indicate significant differences (*P* < 0.05) between chewing time for each sample. No significant difference was reported at 0 chew between samples. YAN was used as a reference and not included in the statistic.

Coatings, salt concentration, and the number of chews influenced the salivary conductivity value. During the in vivo study, there was a slight and gradual increase in salivary conductivity in the mouth when chewing was performed with no noodles in the mouth (blank) (Fig. 3). Saliva is almost 99% water, organic and inorganic substances, comprising proteins and electrolytes like potassium, calcium, chloride, magnesium, bicarbonate and phosphate^41^. These electrolytes contribute to salivary conductivity (blank). The gradual increase elucidated baseline variation in salivary composition, including the change in salivary protein concentration and saliva dilution due to subsequent salivary production. Chewing with unsalted noodles displayed a similar pattern with slightly higher saliva conductivity than blank ones (Fig. 3). It was probably due to the leaching of the *kansui* reagent from the soluble and conductive noodles.

Mastication leads to the disintegration of the noodle structure, increasing the salivary surface area of the food, and promoting the dissolution of taste compounds in the saliva^42^. Salivary α-amylase breaks down starches into soluble maltoses and dextrins, and lingual lipase breaks down a small percentage of dietary triglycerides^43^. These changes increase the conductivity values. The total salivary conductivity was in the order of HC-Na30 > SC-Na30 > HC-Na20 > SC-Na20 > HC-Na10 > SC-Na10 > HC-Na0 > SC-Na0. The results were in expectation since noodles with 30% salt coating comprised the highest quantity of salt compared to other noodles. Thus, the conductivity values were generally attributed to the salt released from the coatings. The salivary conductivity values of all noodles were significantly highest (*P* < 0.05) at 20 chewing cycles, possibly due to the full salt release from the noodles.

Moreover, the composition of saliva is greatly affected by the salivary flow rate. When masticatory, gustatory, or pharmacological stimulation increases salivary flow rate, the percentage of salivary electrolyte (Na^+^, Cl^−^, Ca^2+^, and HCO_3_^−^) increases while K^+^ levels decrease^44^. These electrolytes contribute to salivary conductivity. Salt (NaCl) produces Na+ and Cl− ions when it dissolves in water. These ions contain electrical current and can affect conductivity measurement. Therefore, a solution’s ion concentration positively correlates with its conductivity. In this study, salivary conductivity rises with increasing salt coating.

#### Salivary pH

**Fig. 4:**
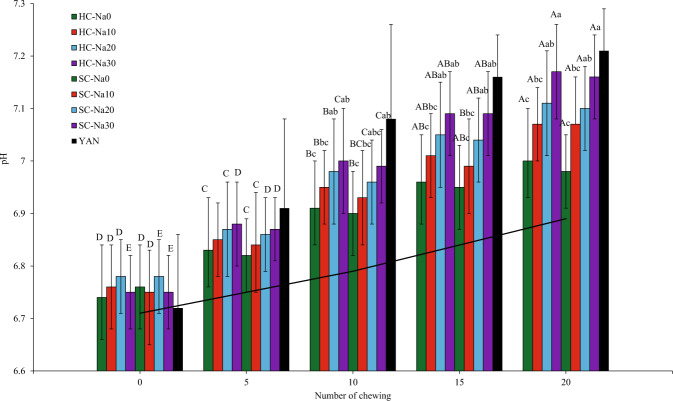
pH values of saliva after chewing different types of noodles at 0, 5, 10, 15, and 20 chews. Error bars indicate mean values ± standard deviations (*n* = 10) values. [—] Saliva conductivity after chewing no noodles (blank) with a fake chewing action. Different lowercase letters indicate significant differences (*P* < 0.05) between samples for each number of chewing. Different uppercase letters indicate significant differences (*P* < 0.05) between chewing time for each sample. No significant difference was reported at 0 and 5 chews between samples. YAN was used as a reference and not included in the statistic.

The pH values of the saliva in the blank chews followed a similar trend as the salivary conductivity in the blank chew (Fig. 4). Salivary pH values collected during chewing may be advantageous in explaining structural deviations and release of *kansui* during chewing. The slight and gradual increase in salivary pH values resulted from saliva production. The impacts of salt coatings and the number of chews on salivary pH are demonstrated in Fig. 4. The overall pH was in the following order: HC-Na30 > SC-Na30 > HC-Na20 > SC-Na20 > HC-Na10 > SC-Na10 > HC-Na0 > SC-Na0. The salivary pH values of all samples ranged between 6.74 and 7.21, and they peaked at 20 chews. The salt concentration in the saliva was the highest at this point and could stimulate saliva production. The normal pH of saliva is 6.0–7.0^44^. Specific salivary components are responsible for the buffering capacity and maintain the pH of the saliva at around 7. The phosphates affect the unstimulated saliva, which is a weaker buffer, while additional bicarbonate ions and histidine-rich peptides contribute to the stronger buffering ability of the stimulated saliva. The salivary flow rate strongly influences the concentration of bicarbonate in saliva, the salivary pH, and the buffer capacity. They accumulate when the salivation accelerates and vice versa^43^. The mechanical action and salt increased the salivary flow and, consequently, the pH. Hence, the highest salivary pH values were observed in HC-Na30 and SC-Na30 noodles.

The pH of saliva from commercial YAN is higher than any samples. It was likely due to the higher salt content of commercial YAN and increased salivation. Chewing carbohydrates in the oral cavity lowered the pH. Enamel erosion occurs when the oral pH is below pH 5.5. Saliva uses bicarbonate (HCO_3_^−^) and phosphate buffering capacity to control the oral pH between 6.8 and 7.8. Bicarbonate and phosphate play a critical role in the cyclic approaches of demineralisation and remineralisation that ward off dental caries^45^. L* values decreased during mastication (data not shown). As the mastication proceeded, salt coatings and food particles were released from the noodles into the saliva, thus reducing L* values. The lowest L* values were reported at 20 chews when the saliva comprised the most food particles and salt coatings. Noodles with 30% salt coatings exhibited higher L* values among all samples.

### Shelf life analysis

#### Microbial changes

YAN has a short shelf life and usually lasts a few days owing to its high moisture with rich nutrient content^46^. Hence, YAN must be refrigerated to avoid microbial deterioration and discolouration during storage. Fresh noodles spoilage caused high waste in the food industry and became a possible cause of foodborne illness^47^. Therefore, TPC, coliform counts, Y & M counts, pH, and L* values were selected to indicate fresh noodle spoilage.

The microbial limit of TPC for ready-to-eat food is 5 log CFU/g as specified in microbiological guidelines issued by Food Quality Control, Ministry of Health Malaysia (MOH)^48^ and Food and Environmental Hygiene Department of Hong Kong^49^. The microbial limit (coliforms) for noodles is 2 log CFU/g according to Stannard^50^. The Y&M counts limit for noodles is 5 log CFU/g based on the Australian Standard^51^. The effect of TPC, coliform counts, and Y&M counts of fresh HC-Na, SC-Na, HC-Na10, and SC-Na10 stored at 4 and 25 °C on different storage days are presented in Tables 3 and 4.

**Table 3:** Total plate counts (TPC), coliform, and yeast and mould (Y&M) changes of fresh noodles during storage at 4 °C.

	Storage time (day)				
	Day 0	Day 8	Day 15	Day 22	Day 29
TPC (log CFU/g)					
HC-Na0	2.5 ± 0.1^Ea^	5.1 ± 0.1 ^Da^	7.2 ± 0.2^Ca^	9.5 ± 0.1^Ba^	10.7 ± 0.2^Aa^
HC-Na10	2.2 ± 0.1^Eb^	4.2 ± 0.1^Db^	5.7 ± 0.1^Cb^	8.8 ± 0.1^Bb^	9.7 ± 0.1^Ab^
SC-Na0	2.5 ± 0.1^Ea^	5.2 ± 0.2 ^Da^	6.9 ± 0.1^Ca^	9.6 ± 0.1^Ba^	10.6 ± 0.1^Ac^
SC-Na10	2.1 ± 0.1^Eb^	4.2 ± 0.1^Db^	5.7 ± 0.2^Cb^	8.7 ± 0.1^Bb^	9.8 ± 0.1^Ab^
YAN	2.4 ± 0.1	4.4 ± 0.1	5.3 ± 0.1	8.8 ± 0.1	9.5 ± 0.1
Coliform (log CFU/g)					
HC-Na0	0.8 ± 0.1^Ea^	1.1 ± 0.1 ^Dab^	1.3 ± 0.1^Ca^	1.6 ± 0.1^Ba^	1.9 ± 0.1^Aa^
HC-Na10	0.7 ± 0.1 ^Da^	0.9 ± 0.1^Db^	1.1 ± 0.1^Cb^	1.4 ± 0.1^Ba^	1.7 ± 0.1^Aa^
SC-Na0	0.8 ± 0.1 ^Da^	1.1 ± 0.1^Ca^	1.3 ± 0.1^Ca^	1.6 ± 0.1^Ba^	1.9 ± 0.1^Aa^
SC-Na10	0.7 ± 0.1 ^Da^	0.9 ± 0.0^Cb^	1.1 ± 0.1^Cb^	1.4 ± 0.1^Ba^	1.7 ± 0.1^Aa^
YAN	0.6 ± 0.1	0.8 ± 0.1	0.9 ± 0.1	1.2 ± 0.1	1.5 ± 0.1
Y & M (log CFU/g)					
HC-Na0	1.4 ± 0.1^Ea^	3.8 ± 0.1 ^Da^	6.0 ± 0.1^Ca^	8.0 ± 0.1^Ba^	9.2 ± 0.1^Aa^
HC-Na10	1.2 ± 0.1^Eb^	3.5 ± 0.1^Db^	4.9 ± 0.1^Cb^	7.7 ± 0.2^Bb^	8.7 ± 0.1^Ab^
SC-Na0	1.4 ± 0.1^Ea^	3.9 ± 0.1 ^Da^	5.9 ± 0.1^Ca^	7.9 ± 0.1^Bab^	9.1 ± 0.1^Aa^
SC-Na10	1.2 ± 0.1^Eb^	3.4 ± 0.1^Db^	4.8 ± 0.1^Cb^	7.6 ± 0.1^Bb^	8.7 ± 0.1^Ab^
YAN	1.3 ± 0.1	3.1 ± 0.1	4.4 ± 0.1	7.1 ± 0.1	8.1 ± 0.1

Results are presented as mean values ± standard deviation (*n* = 3) values. Different uppercase letters indicate significant differences (*P* < 0.05) between storage periods for each sample. Different lowercase letters indicate significant differences (*P* < 0.05) between samples for each storage period. YAN was used as a reference and not included in the statistic.

**Table 4:** Total plate counts (TPC), coliform, and yeast and mould (Y&M) changes of fresh noodles during storage at 25 °C.

	Storage time (day)				
	Day 0	Day 1	Day 2	Day 3	Day 4
TPC (log CFU/g)					
HC-Na0	0.9 ± 0.1^Ea^	6.0 ± 0.1 ^Da^	8.8 ± 0.1^Ca^	11.2 ± 0.2^Ba^	12.0 ± 0.1^Aa^
Hc-Na10	0.9 ± 0.1^Eb^	5.2 ± 0.1^Db^	8.0 ± 0.1^Cb^	9.4 ± 0.1^Bb^	10.1 ± 0.1^Ab^
SC-Na0	1.0 ± 0.2^Ea^	6.0 ± 0.1 ^Da^	8.7 ± 0.1^Ca^	11.0 ± 0.1^Ba^	11.9 ± 0.1^Aa^
SC-Na10	0.9 ± 0.1^Eb^	5.3 ± 0.1^Db^	8.0 ± 0.1^Cb^	9.3 ± 0.1^Bb^	10.0 ± 0.1^Ab^
YAN	0.8 ± 0.1	4.4 ± 0.1	6.9 ± 0.1	8.0 ± 0.1	9.2 ± 0.1
Coliform (log CFU/g)					
HC-Na0	0.8 ± 0.1^Ea^	5.8 ± 0.1 ^Da^	7.0 ± 0.1^Ca^	8.2 ± 0.0^Ba^	8.9 ± 0.1^Aa^
HC-Na10	0.7 ± 0.1^Eb^	5.0 ± 0.1^Db^	6.5 ± 0.1^Cb^	7.7 ± 0.1^Bb^	8.0 ± 0.1^Ab^
SC-Na0	0.8 ± 0.1^Ea^	5.9 ± 0.1 ^Da^	7.1 ± 0.1^Ca^	8.3 ± 0.1^Ba^	9.0 ± 0.1^Aa^
SC-Na10	0.7 ± 0.1^Eb^	5.0 ± 0.1^Db^	6.6 ± 0.1^Cb^	7.6 ± 0.1^Bb^	8.0 ± 0.1^Ab^
YAN	0.6 ± 0.1	4.5 ± 0.1	5.8 ± 0.1	6.8 ± 0.1	7.5 ± 0.1
Y & M (log CFU/g)					
HC-Na0	0.1 ± 0.1^Ea^	5.4 ± 0.1 ^Da^	7.3 ± 0.1^Ca^	8.3 ± 0.1^Ba^	9.0 ± 0.1^Aa^
HC-Na10	0.0 ± 0.1^Ea^	4.8 ± 0.1^Db^	6.2 ± 0.1^Cb^	7.6 ± 0.1^Bb^	8.1 ± 0.1^Ab^
SC-Na0	0.1 ± 0.0^Ea^	5.3 ± 0.1^Da^	7.4 ± 0.1^Ca^	8.2 ± 0.0^Ba^	9.0 ± 0.1^Aa^
SC-Na10	0.1 ± 0.0^Ea^	4.7 ± 0.1^Db^	6.3 ± 0.1^Cb^	7.6 ± 0.0^Bb^	8.1 ± 0.1^Ab^
YAN	0.1 ± 0.1	4.3 ± 0.1	5.3 ± 0.1	7.3 ± 0.1	7.7 ± 0.1

Results are presented as mean values ± standard deviation (*n* = 3) values. Different uppercase letters indicate significant differences (*P* < 0.05) between storage periods for each sample. Different lowercase letters indicate significant differences (*P* < 0.05) between samples for each storage period. YAN was used as a reference and not included in the statistic.

Microbial growth and proliferation significantly impact food deterioration^46^. Salt concentration, storage time, and the interaction between salt concentration and storage time (excluding Coliform at 4 °C) significantly influenced the microbial results at 4 and 25 °C (*P* < 0.05). Samples spoilage was minimised at 4 °C, and the shelf life was increased by limiting the microbial growth in the samples. During storage at 4 °C, the TPC, coliform counts, and Y & M counts in each fresh noodle increased moderately. HC-Na0 and SC-Na0 achieved the microbial limit of TPC by Day 8, while the TPC of HC-Na10 and SC-Na10 were above 5 log CFU/g by Day 15 (Table 3). All samples showed coliform counts lower than the microbial limit (2 log CFU/g) during storage at 4 °C (Table 3). The Y & M counts of HC-Na0 and SC-Na0 noodles exceeded the limit (5 log CFU/g) by Day 15 (Table 3). The approximate shelf lives of HC-Na0 and SC-Na0 were less than 8 days when stored at 4 °C, while that of HC-Na10 and SC-Na10 were longer than 8 days. It could be due to applying a 10% salt coating, which prevents food spoilage by regulating enzyme activity and reducing microbial growth^20^. Ma et al.^46^ reported that the shelf life of fresh noodles was 1–2 days.

Table 4 shows that the microbial counts of all samples surpassed the microbial limits (5 and 2 log CFU/g) of TPC and coliforms on Day 1 when stored at 25 °C. For Y & M, HC-Na0 and SC-Na0 exceeded the microbial restriction on Day 1, while HC-Na10 and SC-Na10 crossed the microbial limit on Day 2. Nevertheless, all samples had a shelf life of less than one day (Table 4). The TPC, coliform counts, and Y & M counts in each fresh sample increased significantly during storage at 25 °C. Li et al.^52^ found that the rapid microbial growth in noodles during the first 24 h was influenced by the rich nutrient composition of the noodles and the storage conditions. Hermetically sealed noodles stored at room temperature have a higher microbial growth rate because they are high moisture products prone to microbial spoilage and decomposition, promoting a short shelf life. Tan et al.^20^ illustrated that the fresh white salted noodles stored at 25 °C had a shorter shelf life than those held at 4 °C. They also noted that the bacteria growth rate was higher at 10–60 °C. Ma et al.^46^ stated that bacteria, moulds, and yeast usually spoil raw, fresh wheat-based foods. The leading determinant contributing to the increase of TPC in fresh noodles might be the high original TPC (10^4^ CFU/g) in wheat flour^53^. Bacterial proliferation accelerated at room temperature and generated much noodle spoilage and waste. The TPC, coliform counts, and Y & M counts of all fresh samples in the current study were higher than those of fresh commercial YAN.

Rapid proliferation of microorganisms in fresh noodles occurred faster when stored at 25 °C compared to 4 °C. Therefore, a temperature of 4 °C is the most suitable to maintain fresh noodles’ shelf life. Microbial growth and proliferation could be inhibited at this temperature^20^. A salt concentration of 10% can prolong the shelf life of fresh YAN.

#### pH changes

**Fig. 5:**
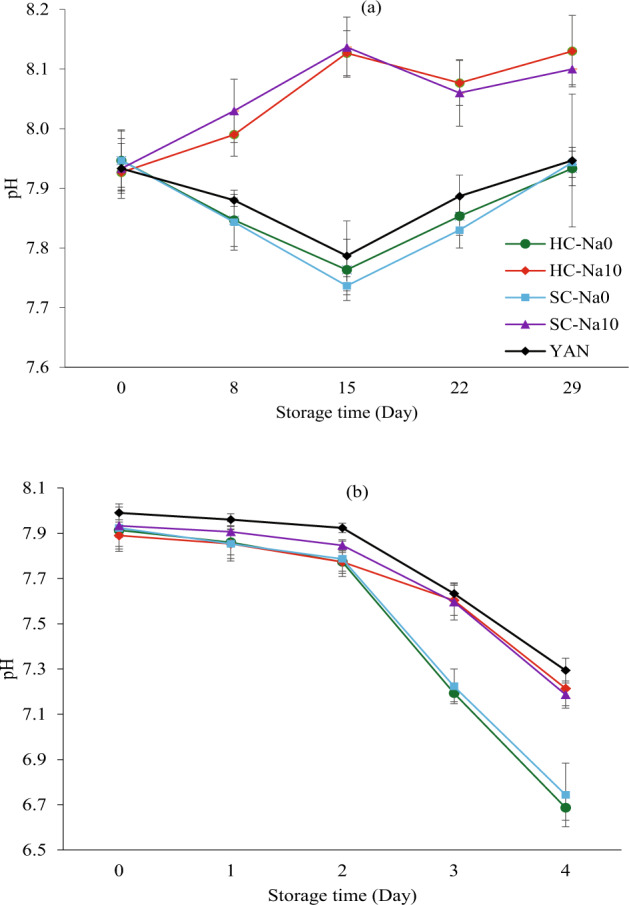
pH changes of different types of noodles during storage. Results are presented as mean ± standard deviation (*n* = 3) values. **a** pH changes of HC-Na0, HC-Na10, SC-Na0, SC-Na10 and YAN during storage at 4 ℃. **b** pH changes of HC-Na0, HC-Na10, SC-Na0, SC-Na10 and YAN during storage at 25 ℃.

Salt concentration, storage time, and interaction between salt concentration and storage time significantly influenced the pH values at 4 °C and 25 °C (*P* < 0.05). The pH changes of fresh noodles are listed in Fig. 5. The initial pH of all noodles was between 7.89 and 7.95. Tan et al.^20^ reported that pH 6–8 is the optimum pH for bacteria growth. At 4 °C, the pH of HC-Na0 and SC-Na0 decreased from Day 0 to Day 15 and increased from Day 15 to Day 29. The pH of HC-Na10 and SC-Na10 increased from Day 0 to Day 15 and decreased from Day 15 and Day 29. The increase in pH, as demonstrated by HC-Na10 and SC-Na10 from Day 0 to Day 15 and HC-Na0, SC-Na0, and YAN from Day 15 to Day 29, illustrated low acid levels in noodles. Li et al.^3^ observed that some essential compounds, including amine and ammonia, were released because of the decomposition of proteins in fresh noodles. The presence of these substances could neutralise and stabilise the fermentative acids and lead to an increase in pH. Proteolytic bacteria and moulds released ammonia from proteins^54^. The decrease in pH values of HC-Na0, SC-Na0, and YAN from Day 0 to Day 15 and HC-Na10 and SC-Na10 from Day 15 to Day 29 could be attributed to microorganism proliferation. Ma et al.^46^ found that pH reduction signified fresh noodle deterioration. Li et al.^52^ stated that pH lowering caused by microbial growth was typical because microorganisms consumed the abundant carbohydrates in food to produce acids and alleviated the pH. Fermentation is one of the critical spoilages of fresh noodles.

At 25 °C, the pH values of all noodle samples declined continuously from Day 0 to Day 4 (Fig. 5b). The decrease in pH indicated high acid concentration in noodles. The noodle samples were most susceptible to deterioration and microorganism attack at 25 °C. Similar results were reported by Guo et al.^55^, where the pH values of noodles declined during storage at 25 °C for 5 days. They explained that the acid changes were associated with microbial metabolism. Moreover, the metabolism of carbohydrates by microorganisms led to the production of organic acids such as citric acid, and the metabolism of lipids results in the formation of lactic acid. Fresh noodles in this study had a lower pH during storage than fresh commercial YAN.

#### L* values changes

Consumers opted for bright or creamy white fresh noodles without darkening or discolouration^46^. Salt yielded noodles with white or creamy white colour^32^. Ye at al.^56^ indicated a positive correlation between salt concentration (0 to 2%) and the lightness of noodles. The CIELab L* value reflects the darkening of noodles, showing diffuse light reflectance (scattering) and light absorption^47^.

**Fig. 6:**
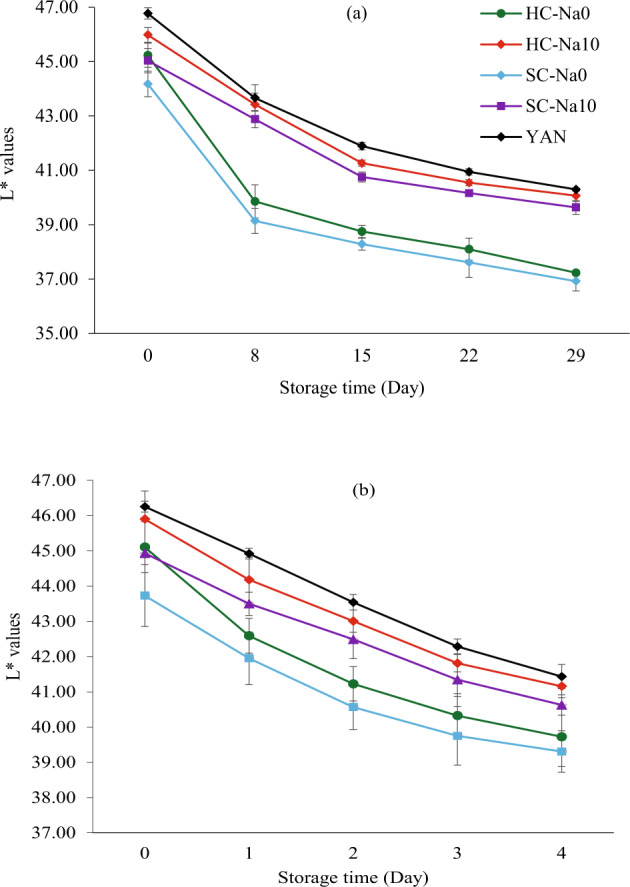
L* values changes of different types of noodles during storage. Results are presented as mean ± standard deviation (*n* = 3) values. **a** L* values changes of HC-Na0, HC-Na10, SC-Na0, SC-Na10 and YAN during storage at 4 °C. **b** L* values changes of HC-Na0, HC-Na10, SC-Na0, SC-Na10 and YAN during storage at 25 °C.

Salt concentration and storage time influenced the L* values at 4 and 25 °C significantly (*P* < 0.05), and the interaction between salt concentration and storage time influenced the L* values at 4 °C significantly (*P* < 0.05). The L* values of all noodle samples reduced with increasing storage time at 4 °C from Day 0 to Day 28 (Fig. 6a) and at 25 °C from Day 0 to Day 4 (Fig. 6b). It could be due to the enzyme polyphenol oxidase (PPO), which was involved in noodles darkening^46^. The autooxidation of phenolic compounds could play a role in darkening as well. Li et al.^47^ pointed out that fresh noodles usually darken with decreasing L* values in the first 24 h, especially 0–4 h. Asenstorfer et al.^37^ suggested that both non-enzymatic and enzymatic reactions could involve browning. They also concluded that the vital process in non-enzymatic browning in YAN was induced by oxidation between protein, specifically tyrosine and glutenin. Moreover, molecular refraction and dispersion on fresh noodles’ outer and inner sides could influence the L* values. The protein molecules and gluten network might change the structure during storage and reduce the reflectivity and L* values^20^. The L*values of all the fresh noodles in this study were lower than the L* of fresh commercial YAN during storage.

A temperature of 4 °C could prolong the shelf life of fresh noodles more than room temperature (25 °C). Al-Tayyar et al.^57^ illustrated that applying edible coatings to the surface of food can extend its shelf life. They also explained that edible coatings, often composed of food additives or antimicrobial agents such as spices, nutrients, flavourings, colours, and anti-browning agents, became increasingly essential to diminish the negative impact of food processing. The application of HC and SC in this study could meet the consumer demand for healthy, nutritious, and natural food.

To the best of our knowledge, salt coatings have not been previously used for any type of food. This study can be a significant step forward in reducing salt content in food and improving public health. In general, structural breakdown behaviours, salt-release properties, taste, and saltiness perception of HC-Na30 and SC-Na30 were close to those of YAN, even though their final salt content was less than 50% of YAN. This observation suggests that concentrating salt on the surface of noodles enhanced the release of salt during chewing, as the structure was broken down and improved saltiness perception. Coating with 10% salt is the most preferred in terms of structural breakdown and texture. Noodles without salt had a shelf life of fewer than 8 days under 4 °C. HC-Na10 and SC-Na10 had a shelf life of more than 8 days if they were stored at 4 °C since refrigeration storage at 4 °C could decelerate the microbiological and pH changes than the storage at 25 °C. These findings support that 10% salt coating could improve the shelf life of fresh noodles. Applying salt-coating technology without impairing YAN’s sensory perception and structural properties is possible to form low-salt noodles with an enhanced shelf life.

## Methods

### Materials

The essential ingredients for the noodle preparation (wheat flour, salt, *kansui* reagent) were purchased from Tesco Stores (M) Sdn. Bhd. (Georgetown, Malaysia). The high amylose corn starch used in this investigation was Hylon^TM^ VII (National Starch and Chemical Limited, Manchester, UK). Semperfresh^TM^ was acquired from Xeda International S.A., France. The reagents and chemicals used in the formulation of the artificial saliva and analysis were of analytical grade. Xanthan gum and α-amylase (type VI-B from Porcine Pancreas) were purchased from Sigma Chemical Co. (St. Louis, US). Sodium hydrogen carbonate, potassium chloride, sodium chloride, and dipotassium hydrogen phosphate (anhydrous) calcium chloride dehydrate were purchased from System Sdn. Bhd. (Selangor, Malaysia). Other chemicals used in this study (analytical grade) were purchased from Sigma Aldrich. All experiments were conducted with deionised water.

### Preparation of fresh unsalted yellow alkaline noodles (YAN)

Fresh unsalted yellow alkaline noodles were prepared according to the method described by Yeoh et al.^26^. Unsalted noodles consisted of 100 g wheat flour (74% carbohydrate, 9% protein, 1% fat), 50 g deionised water and 1 g *kansui* (9:1 sodium and potassium carbonate). The unsalted noodles were divided into 2 groups: (1) HYLONTM-coated (HC) and (2) SemperfreshTM-coated (SC). The formulation of the coated noodles is demonstrated in Table 5. A sample of commercial YAN purchased from a local supermarket was used as a reference for all analyses except shelf life analysis.

**Table 5:** Formulation and designation of coated-noodle samples.

Ingredients (g)	Types of noodles							
	HC-Na0	HC-Na10	HC-Na20	HC-Na30	SC-Na0	SC-Na10	SC-Na20	SC-Na30
Hylon VII	15	15	15	15	-	-	-	-
Semperfresh	-	-	-	-	5	5	5	5
NaCl	0	10	20	30	0	10	20	30

“-” denotes the absence of the ingredient in the formulation. Na0, 0% salt, Na10, 10% salt, Na20, 20% salt, Na30, 30% salt.

*NaCl* sodium chloride, *HC* Hylon-coated, *SC* semperfresh-coated.

### Preparation of Hylon-coated noodles (HC noodles)

According to Yeoh et al.^26^, the preparation of HYLON^TM^-coated noodles was conducted. A 15% (w/v) slurry was prepared by placing 15 g of HYLONTM VII starch in 100 mL volumetric flasks. Salt (NaCl) 10, 20, and 30% (w/v) were added to the flask according to Table 5. Sufficient deionised water was added to bring the final volume to 100 mL. The resulting slurry was boiled in a boiling water bath with stirring for 30 min before being autoclaved at 110 °C for 30 min to obtain gelatinisation. The gelatinised starch solution was put in a beaker in a water bath (95 °C). The unsalted noodles were immersed in the starch solution for 1 min and air-dried for 6 h at 30 °C using an IN110 incubator (Memmert, Germany).

### Preparation of SemperfreshTM-coated noodles (SC noodles)

The preparation of SC noodles was conducted according to the method described by Yeoh et al.^26^. A 5% (v/v) solution was prepared by adding 5 mL each of Semperfresh^TM^ into a 100 mL volumetric flask. Salt (NaCl) 10, 20, and 30% (w/v) were added to the flask according to Table 5. Adequate deionised water was added to bring the final volume to 100 mL. The solution was stirred magnetically. The noodles were immersed in the SemperfreshTM solution for 1 min, then air-dried for 6 h at 30 °C using an IN110 incubator (Memmert, Germany).

### Preparation of fresh commercial YAN

Fresh commercial YAN consists of 100 g of wheat flour, 50 g of deionised water, 1 g of *kansui* reagent (9:1 sodium and potassium carbonate), and 1.75 g of salt was prepared according to Yeoh et al.^58^. The salt content (1.75 g) was selected based on the sodium result of commercial YAN purchased from a local supermarket^26^.

### Determination of noodle optimum cooking time

The noodle optimal cooking time for each sample was determined according to the AACC method 66–50^59^. In a saucepan, noodle samples (15 g) were cooked in boiling deionised water (at a ratio of 1:20, one-part noodles to 20 parts water). The optimal cooking time was defined when the white core in the central part of the noodle strand disappeared when the noodle was squeezed between two transparent glass plates.

### Determination of sodium in noodles by flame atomic absorption spectrometry (FAAS)

The sodium in noodles was determined according to the method described by Yeoh et al.^26^. A closed microwave system Multiwave 3000 (Anton Paar, Germany) was used to digest the freeze-dried samples (200–250 mg). The absorbance of all samples was measured using an AA-7000 atomic absorption spectrophotometer (Shimadzu, Japan) at a wavelength of 589 nm. Three replicates were made for each type of noodle.

### Sensory evaluation

The sensory evaluation was performed at the sensory laboratory of the School of Industrial Technology, Universiti Sains Malaysia, Penang. The sensory panellists consisted of 30 food science undergraduate and postgraduate students (20–40 years old males and females) from the Food Technology Division at Universiti Sains Malaysia who had taken a course in sensory science. All participants were provided written informed consent. They were frequent consumers of noodles and well-informed before the sensory evaluation. HC noodles, SC noodles, and commercial YAN were cut into 5 cm pieces and then cooked in boiling water at a ratio of 1:10 (one-part noodles in 10 parts water) at the optimum cooking time using a stainless-steel pot and gas stove. The samples were then stored in a covered container before being served with soup to the panellists in a 3–4 g portion on trays labelled with random three-digit numbers and in random order. All samples were evaluated on a seven-point hedonic scale (7 = like extremely, 4 = neither life nor dislike, 1 = dislike extremely)^23^. The samples were evaluated for taste and saltiness.

### Structural breakdown analysis

Structural breakdown analysis was conducted according to Tan et al.^20^ using a multiple extrusion cell (MEC) attached to a texture analyser (model TA-TX2, Stable Micro Systems, Surrey, UK) fitted with a 30 kg load cell. Twenty grams of the noodle samples were placed in the sample vessel containing 10 mL of artificial saliva. Three replicates were made for each type of noodle. The formula of the artificial saliva was NaHCO_3_ (5.208 g), K_2_HPO_4_ (1.241 g), NaCl (0.877 g), KCl (0.447 g), CaCl_2_·2H_2_O (0.441 g), xanthan gum (0.920 g) and α-amylase (2,000,000 U) in 1 L of distilled water. The pH of the artificial saliva was adjusted to 7.0 by 0.1 M HCl. The device setting: Mode, measure force in compression; Option, cycle until count; Test Speed, 10 mm/s; Post-Test Speed, 5 mm/s; Distance, 95 mm; Count, 20 cycles; Data Acquisition Rate, 2 pps. A force (N) vs time (s) plot was constructed, and the data was plotted into a single exponential decay equation in Eq. 1.

1 $$W_{(n)} = w_{\rm{inf}} + w_1{\rm{EXP}}\left( { - \frac{n}{{n_1}}} \right)$$

where *W(n)* represents work done during each extrusion cycle (*n*), *w*_inf_ is work per extrusion after a large (infinite) number of extrusions, *w*_*1*_ defines the amount and strength of degraded noodle structure, and *n*_*1*_ defines the decay rate of work per extrusion with an increasing number of extrusions.

### Digital microscope image analysis

The cooked noodles were prepared according to their optimum cooking time. The image of fresh raw and cooked HC and SC noodles with selected salt concentrations (0%, 10%, and 30% salt) were observed using a digital microscope (VHX-7000, Keyence, Japan). Commercial YAN was used as a reference. Noodles were cut by a surgical blade and observed at ×100 magnification.

### Microstructure analysis

The microstructure of all raw and cooked HC and SC noodles was observed with a scanning electron microscope (SEM) (Quanta FEG 650, Fei, USA)^39^. Noodles were coated with Pt-Pd before viewing the cross-section of the noodles at ×100 magnification. Commercial YAN was used as a reference.

### Determination of salivary conductivity and pH: an in vivo study

The experiment was approved by the University human ethics committee (code number: USM/JEPeM/17050264, Jawatankuasa Penyelidikan Manusia USM (JEPeM)). All participants were provided written informed consent.

The method was adopted by Boisard et al.^60^ and Tian & Fisk^25^ with modifications. The subjects recruited are generally healthy, non-smokers, and between 26-37 years of age. They were discouraged from participating in this study if they had a gluten allergy, were ill, smoked, or consumed alcoholic beverages. Ten subjects (5 females and 5 males) from the School of Industrial Technology, Universiti Sains Malaysia, Pulau Pinang, had to chew the noodles a defined number of times and according to their usual way while sitting. A total of 12 one-hour sessions were performed by each subject (4 sessions in the same week, each session at the same time of day). During each session, 2–3 experiments were conducted in a balanced order. Ten replicates were performed for nine samples (HC noodles, SC noodles, and commercial YAN). Ten subjects were asked to chew a blank sample (no noodles), whereby they were asked not to chew anything in their mouth for 0, 5, 10, 15, and 20 chews. Then their saliva (approximately 0.2 mL) was collected using a modified method (saliva collection with Lashley cup or Carlson-Crittenden collector, adapted from Bellagambi et al.^61^). Modified scalp vein set 23G sterile PVC tubing with 0.63 mm × 19 mm (Top Winged Infusion Set, Malaysia) was attached to a 3 mL sterile syringe (Terumo, Philippines) to extract saliva from the mouth. Commercial YAN was used as a reference.

Saliva was collected at 5 chewing intervals: before eating the noodles (coded C0) and after 5, 10, 15, and 20 chewing cycles (coded C5, C10, C15, C20). An interval of 40 s was imposed between each sample. The human subjects were asked to clean their mouth with deionised water before the experiment to ensure there were no noodle particles in their mouth. They had to avoid taking food and liquid (except water) and chewing gum before 30 min of this study and connect the tube to the syringe and insert it into their mouth. They were instructed to use the syringe and tube to extract their saliva before chewing the noodles and immediately after 5, 10, 15, and 20 chews. The saliva samples were directly placed on ice.

Saliva samples were weighted, diluted 1000× with deionised water, and vortexed for 1 min before analysis. Saliva conductivity was measured with a sensION+ EC5 handheld conductivity metre and probe (Hach, Colorado, USA). The conductivity probe was placed in the saliva sample solution to estimate the salt level. pH measurement of saliva samples was conducted with a Mettler-Toledo Delta 320 pH metre calibrated with a pH 4.0 and 10.0 buffer solution^23^.

### Shelf life analysis

Salt coatings (10%, HC and SC) were selected based on the previous study^26^ in which noodles with 10% salt coating had the highest textural and mechanical parameters, sensory hardness, and springiness significantly. Shelf life analysis was performed according to Tan et al.^20^. Each fresh sample was stored in a polythene bag after preparation, and a fresh commercial YAN containing 1.75 g of salt was prepared in the laboratory to serve as a reference.

The samples were stored in an IN110 incubator (Memmert, Germany) at 25 °C and a GR-M48MP refrigerator (Toshiba, Japan) at 4 °C. Microbial, pH, and colour were selected criteria for spoilage of noodles. Three replicates were made for each type of noodle.

### Microbial changes

The samples were analysed according to Tan et al.^20^ for total plate counts (TPC), yeast and mould counts (Y&M), and coliform counts. Sample (25 g) and sterile peptone water (225 mL) were mixed in a stomacher bag for 2 min. Serial dilutions of 10-1 to 10-6 were made, and 100 µL of the dilution was inoculated into Petri film. Petri films for TPC and coliform counts were incubated at 37 °C for 48 ± 2 h, while Petri films for Y&M counts were incubated at 25 °C for 5 days. All results were expressed in log CFU/g.

### pH changes

The changes in pH of the samples were determined using a Seveneasy pH metre (Mettler Toledo)^20^. Measurements were performed in triplicate, and the results are shown as the average.

### L* values changes

The L* values (lightness) of noodles sheets were determined according to the method described by Tan et al.^20^. The changes in the L* value of each sheet were measured with the CIE 1976 L* colour scale using a colour spectrophotometer (Konica Minolta, CM3500-d, Japan). Each noodle sheet was shaped into pieces of 5 cm (diameter). Measurements were made in triplicate, each at a different location but on the same side of the noodle sheet surface.

### Statistical analysis

Results were expressed as mean ± standard deviation. Data were analysed using SPSS Window, version 26.0 (*P* < 0.05) (SPSS Inc., Chicago, IL, USA). Comparisons of means were performed by analysis of variance followed by Tukey’s test.

## Data Availability

Data sharing is not applicable to the main text. The data supporting the findings of this study are available on request from the corresponding authors.
